# Partial TG6 loss of function causes motor deficits in male mice

**DOI:** 10.1093/hmg/ddag037

**Published:** 2026-06-04

**Authors:** Luisa Donini, Linda Sartori, Anna Barbieri, Alice Migazzi, Sergio Robbiati, Maria Pennuto, Manuela Basso

**Affiliations:** Department of Cellular, Computational and Integrative Biology (CIBIO), University of Trento, via Sommarive 9, 38123 Trento, Italy; Department of Cellular, Computational and Integrative Biology (CIBIO), University of Trento, via Sommarive 9, 38123 Trento, Italy; Department of Cellular, Computational and Integrative Biology (CIBIO), University of Trento, via Sommarive 9, 38123 Trento, Italy; Department of Cellular, Computational and Integrative Biology (CIBIO), University of Trento, via Sommarive 9, 38123 Trento, Italy; Department of Cellular, Computational and Integrative Biology (CIBIO), University of Trento, via Sommarive 9, 38123 Trento, Italy; Model Organism Core Facility (MOF), Department CIBIO, University of Trento, via Sommarive 9, 38123 Trento, Italy; Department of Biomedical Sciences (DBS), University of Padova, via Ugo Bassi 58/B, 35131 Padova, Italy; Veneto Institute of Molecular Medicine (VIMM), via Giuseppe Orus 2, 35129 Padova, Italy; Department of Cellular, Computational and Integrative Biology (CIBIO), University of Trento, via Sommarive 9, 38123 Trento, Italy

**Keywords:** transglutaminase 6, knockdown, mouse model, sexual dimorphism, mouse behavior

## Abstract

Mutations in Transglutaminase 6 (TG6) have been linked to a genetic form of spinocerebellar ataxia, namely SCA35. In recent years, several mutations associated with this disease have been identified. While some of them did not alter TG6 enzymatic activity, others induced a dominant-negative loss-of-function and altered subcellular localization. We previously observed that mutations identified in patients, which showed detrimental effects on neuronal viability in vitro, including mislocalization and activation of the unfolded protein response, were consistently characterized by a loss of TG6 enzymatic function. To investigate this effect *in vivo*, we re-derived *Tgm6* knockout mice from the EMMA repository and performed behavioral characterization. We measured body weight and assessed motor performance using the rotarod, elevated beam/beam balance test, and ladder test, beginning at 1 month of age and continuing through 16 months of age. Here, we report that TG6 loss-of-function impairs motor coordination in male mice, suggesting a sex-specific function for this enzyme.

## Introduction

Among the various SCAs, autosomal Spinocerebellar Ataxia Type 35 (SCA35) is generally characterized by severe symptoms, including cerebellar ataxia, upper and lower-limb ataxia, dysarthria, ocular dysmetria, and tremors. SCA35 has been described with a slow progression and variable onset from adolescence to middle age; rarely, cognitive impairment was also reported [1]. SCA35 was first associated with two missense mutations in the transglutaminase 6 (*TGM6*) gene in a four-generation analysis of a Chinese Han family [2]. Further studies have reported additional mutations observed in apparently healthy individuals, casting doubt on the true pathogenicity of *TGM6* mutations [3]. For example, twelve patients with known pathogenic *TGM6* mutations did not display typical SCA35 symptoms, but congenital myopathy, hypertrophic cardiomyopathy, seizures, and febrile convulsions [4]. Family members carrying *TGM6* mutations were found to be asymptomatic carriers. *TGM6* mutations were also reported in patients with non-neurodegenerative diseases (non-NDDs) [4]. These studies underscore the need for accurate variant classification and a deeper understanding of *TGM6’s* biological functions.

*TGM6* encodes transglutaminase 6 (TG6), a member of the transglutaminase (TG) family. This family comprises nine members: TG1–7, factor XIIIa, and the structural protein, band 4.2 [5]. Except for the last one, all eight members are calcium-dependent enzymes that catalyze cross-linking reactions between the γ-carboxamide end of proteins and the amino residue of primary amines. Beyond the possibility of forming new covalent bonds for peptide chains, transglutaminases can also stabilize protein structures through intramolecular bond formation. The conserved TG structure comprises an N-terminal region, a catalytic domain, and two beta-barrel domains at the C-terminus. A peculiarity of all TGs is that they lack amino glycosylation or disulfide bonds, despite possessing several glycosylation sites and cysteines. Notably, they also do not exhibit a terminal hydrophobic lead sequence, even in secreted TG types [6]. Nevertheless, they can be distinguished by physiological function, specificity, and tissue of expression; they are evolutionarily related: intronic patterns, genomic sequences, protein folding, and catalytic functions are similar within the family [7]. The *TGM6* gene is located on chromosome 20p13–12.2, adjacent to the *TGM3* gene, and its transcript comprises 13 exons and 12 introns. TG6 catalyzes the specific reaction between an L-glutaminyl carboxamide residue and the amino group of L-lysine. The TG6 active site comprises three highly conserved residues (cysteine, histidine, and aspartic acid) that bind the glutamine residue, as well as 12 metal-binding sites that can accommodate up to 3 calcium cofactors [8]. TG6 is mostly expressed in cerebellar neurons and in Purkinje cells [9]. Several mutations in TG6 have been associated with a dominant-negative loss of enzymatic function [9], suggesting that reduced transamidase activity contributes to neuronal degeneration.

Another piece of evidence pointing to a loss of TG6 function as a causative factor in SCA35 is the presence of autoantibodies against TG6 [10]. This event has been linked to gluten sensitivity in a case report of SCA35, in which dietary management with gluten-free foods improved overall limb stiffness and led to a significant decrease in the score on the scale for the assessment of ataxia (SARA) [11].

To determine whether TG6 loss-of-function is sufficient to induce an ataxic phenotype, we characterized a *Tgm6* knockout (TG6 KO) mouse model developed by the EMMA consortium [12]. We observed impaired balance and coordination only in male mice at 10 months of age. This supports the hypothesis that loss of TG6 function plays a role in motor coordination dysfunction, with a sex bias.

## Results

### Reduction of *Tgm6* expression in the mouse model Tgm6 < tm1a from INFRAFRONTIER/EMMA

**Figure 1:**
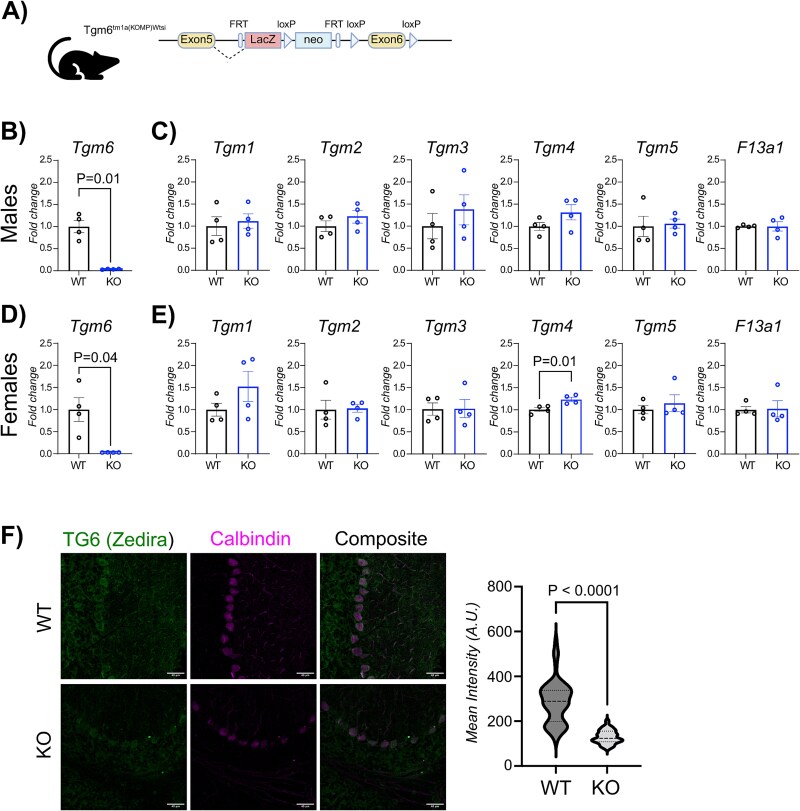
In vivo expression levels of *Tgm6*. (A) Schematic representation of the knockout-first allele tm1a for *Tgm6*. (B-C) *Tgm6* transcriptional levels quantification through qPCR in the cerebellum tissues of male and female TG6 WT/KO mice (males: *n* = 4 WT, 4 *n* = 4 KO; females: *n* = 4 WT, and *n* = 4 KO). Reported data are normalized to *Actb* levels and relativized to WT samples. Graphs, mean ± SEM, Welch’s t test. (D-E) *Tgm1–5* and *F13a1* transcriptional levels quantification through qPCR in the cerebellum tissues of male and female TG6 WT/KO mice (males: *n* = 4 WT, 4 *n* = 4 KO; females: *n* = 4 WT, and *n* = 4 KO). Reported data are normalized to *Actb* levels and relativized to WT samples. Graphs, mean ± SEM, Welch’s t test. F) (left) representative images of immunostaining for TG6 in cerebellar Purkinje neurons, which are marked with calbindin, in mouse brain slices. Scale bar 40 μm. (right) quantification of TG6 signal in calbindin-positive cells for both Tgm6-WT and Tgm6-KO animals (*n* = 1 per genotype). Graphs, mean ± SEM, unpaired two-tailed Student’s *t-*test.

To elucidate the role of *Tgm6* loss-of-function in SCA35 pathogenesis, we characterized a mouse model with reduced *Tgm6* expression, generated by the International Knockout Mouse Consortium (IKMC) and the European Mouse Mutant Archive (EMMA) [13]. Using a targeting cassette between exons 5 and 6 of *Tgm6*, the Consortium generated a ‘Knockout-first allele’ [12] in C57BL/6 N embryonic stem cells [14]. The presence of the targeting cassette results in the production of a truncated transcript, predicted to generate a null allele. This insertion is noted as tm1a (Fig. 1A).

We tested whether mice with Tgm6 < tm1a in both alleles (knockout, KO) showed reduced *Tgm6* mRNA levels compared to wild-type (WT) mice. We collected the cerebellum from adult male and female mice, extracted RNA, and performed real-time (RT) PCR. In both males and females, we observed a reduction of *Tgm6* expression (Fig. 1B-C), suggesting that the insertion of the targeting cassette in the *Tgm6* gene is sufficient to induce a loss of *Tgm6* expression. We also monitored the expression levels of other members of the TGs family, i.e. *Tgm1*, *Tgm2*, *Tgm3*, *Tgm4*, *Tgm5*, and *F13a1*. We observed no alteration in the levels of these other TGs in males, whereas we measured a significant increase in *Tgm4* transcription in females (Fig. 1D-E).

To validate the lowering of TG6 protein expression, we measured TG6 in lysates of cerebella samples. Unfortunately, no commercially available antibodies could specifically detect TG6 (Supplementary Fig. 1A-B). One of the antibodies tested retrieved a band at the correct molecular weight, with prominent expression in astrocytes and not in neurons (Supplementary Fig. 1B-C). However, this is in disagreement with previously published data [8]. To test the specificity of the selected antibody, we transduced primary astrocytes derived from the tm1a mice with an AAV expressing Cre recombinase. Upon Cre recombinase expression, *Tgm6* is deleted, as shown by the real-time (RT) PCR (Supplementary Fig. 1D). Regrettably, a reduction in TG6 protein levels was not observed (Supplementary Fig. 1E), confirming the antibody’s non-specificity in immunoblotting. We then attempted to visualize TG6 expression by immunofluorescence using an antibody we had previously tested [9]. When we compared the WT mice with Tgm6 < tm1a mice, we observed a significant reduction in TG6 expression in Purkinje cells (Fig. 1F).

These results provide evidence that Tgm6 < tm1a mice exhibit reduced TG6 expression and can serve as a suitable model for investigating TG6 loss-of-function.

### Male Tgm6 < tm1a mice display impaired motor coordination

**Figure 2:**
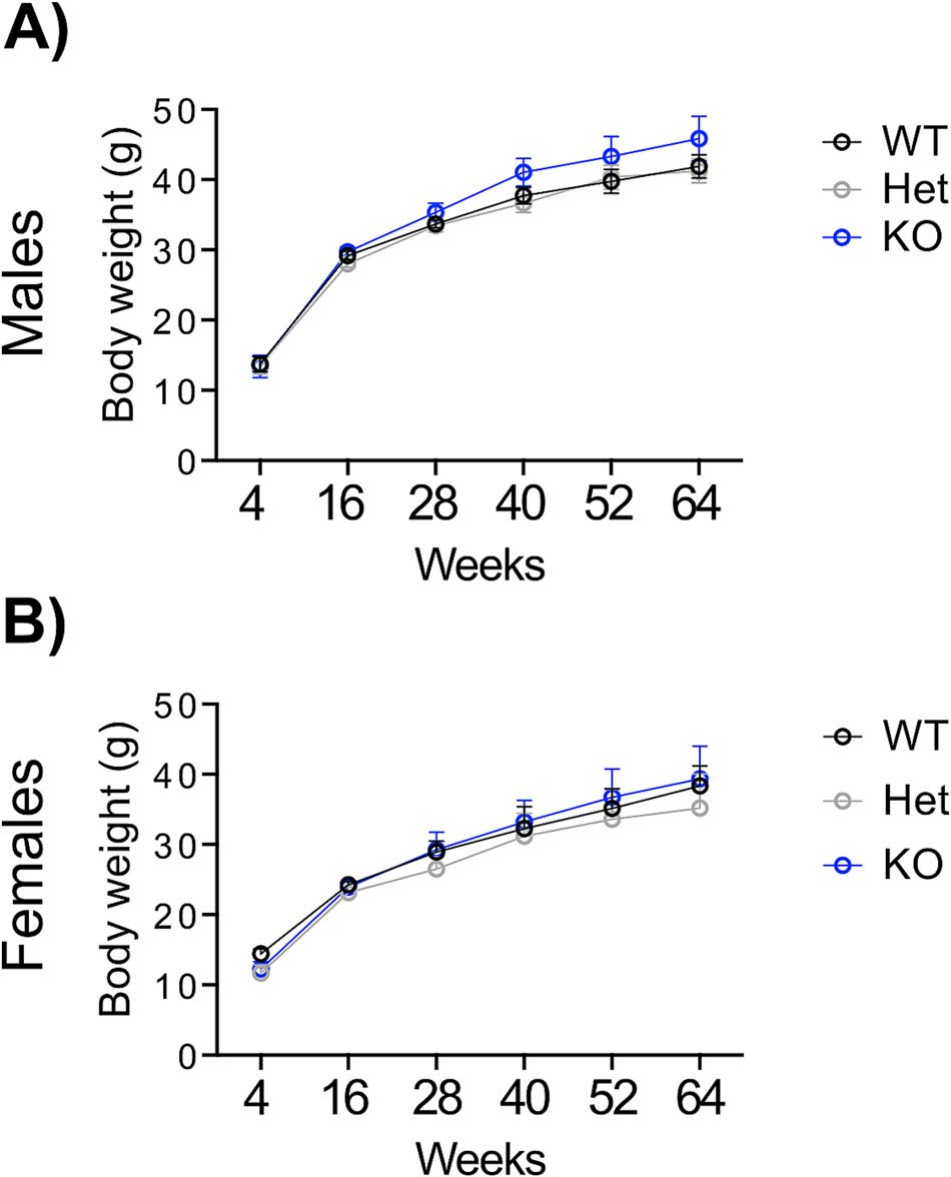
Body weight monitoring. (A-B) body weight for male and female Tgm6-WT/-Het/-KO mice are reported every 4 weeks (males: *n* = 9 WT, *n* = 8 Het, *n* = 6 KO; females: *n* = 5 WT, *n* = 6 Het, and *n* = 7 KO). Graphs, mean ± SEM, mixed-effect analysis, Tukey’s multiple comparisons test.

To evaluate whether *Tgm6* reduction induces an ataxic phenotype in mice, we followed TG6 WT, Het, and KO mice from 4 to 64 weeks of age. To monitor body weight over time, we weighed the mice every week. In Fig. 2, we report the changes in body weight every 4 weeks. TG6 KO male mice showed a non-significant trend of increased body weight compared to WT and Het mice (Fig. 2A), whereas female mice showed similar body weight across all groups (Fig. 2B).

To assess the potential onset of an ataxic phenotype, we employed behavioral tasks, including the balance beam, ladder test, and rotarod. We tested the mice at 1, 4, 7, 10, 13, and 16 months of age.

In the balance beam, mice were instructed to walk on a bar. If the mice were unable to complete the task, a 60-second time limit was arbitrarily recorded.

**Figure 3:**
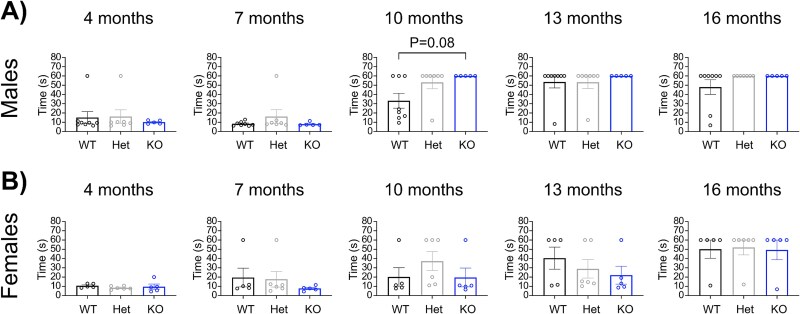
Performance of the mice cohorts in the balance beam test. (A) the time required for male animals to traverse the beam is expressed in seconds (*n* = 8WT, *n* = 7 Het, and *n* = 5 KO). Graph, mean ± SEM, Kruskal-Wallis test followed by Dunn’s post-hoc comparisons at each time point. (B) the time required for female animals to traverse the beam is expressed in seconds (*n* = 5 WT, *n* = 6 Het, *n* = 5 KO). Graph, mean ± SEM, Kruskal-Wallis test followed by Dunn’s post-hoc comparisons at each time point.

Tgm6 < tm1a male mice at 10 months of age showed increased inability to complete the task. The Fisher’s test yielded a p-value of 0.0918, suggesting a trend toward decreased balance (Fig. 3A). Female mice did not show a difference among genotypes (Fig. 3B).

**Figure 4:**
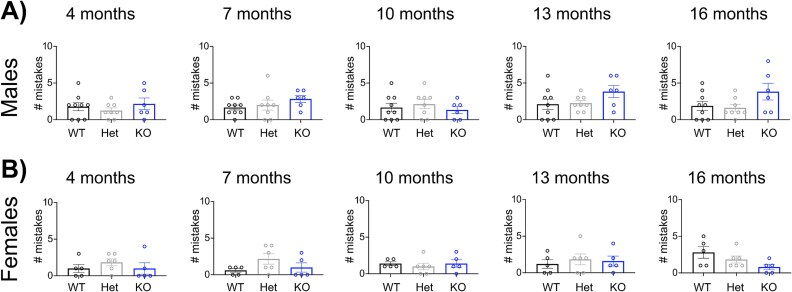
Mouse performance on the ladder test. (A) the total number of mistakes measured during the ladder test was reported for TG6 WT/Het/KO male animals (*n* = 9 WT, *n* = 8 Het, and *n* = 6 KO). Graph, mean ± SEM, Kruskal-Wallis test followed by Dunn’s post-hoc comparisons at each time point. (B) the total number of mistakes measured during the ladder test was reported for TG6 WT/Het/KO female animals (*n* = 5 WT, *n* = 6 Het, *n* = 5 KO). Graph, mean ± SEM, Kruskal-Wallis test followed by Dunn’s post-hoc comparisons at each time point.

To test the ability to place, step, and coordinate forelimbs and hindlimbs, we set up a ladder test. We created a ladder with unequal distribution of the steps. We alternated between short and long distances and tested the mice’s ability to move on them. As a readout of motor coordination impairment, we counted errors. We noticed an increased propensity to make mistakes at 7 and 13 months of age in Tgm6 < tm1a male mice (Fig. 4A). No differences or trends were observed in female mice across genotypes and age (Fig. 4B).

**Figure 5:**
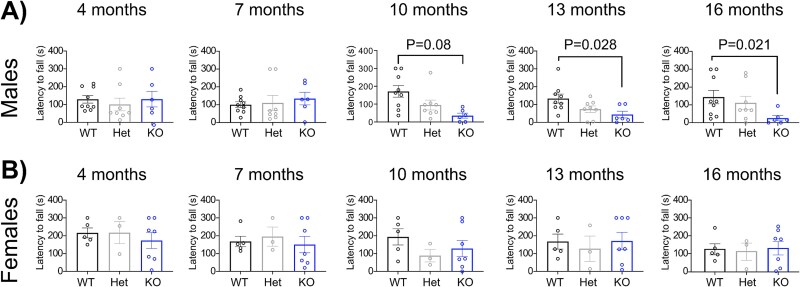
Locomotor performance of TG6 KO mice. (A) Analysis of TG6 WT/Het/KO male mice on the rotarod (*n* = 9 WT, *n* = 8 Het, and *n* = 6 KO). The latency to fall in seconds is reported. Graphs, mean ± SEM, Kruskal-Wallis test followed by Dunn’s post-hoc comparisons. (B) Analysis of TG6 WT/Het/KO female mice on the rotarod (*n* = 5 WT, *n* = 3 Het, and *n* = 7 KO). The latency to fall in seconds is reported. Graphs, mean ± SEM, Kruskal-Wallis test followed by Dunn’s post-hoc comparisons.

Finally, we assessed the locomotor activity with the rotarod test. We employed a constant-velocity protocol to test motor coordination rather than muscle strength or endurance. Tgm6 < tm1a male mice showed a significant reduction in the latency to fall at 10, 13, and 16 months of age (Fig. 5A), while no differences were observed in female mice (Fig. 5B).

Altogether, these results suggest a selective alteration of motor abilities in male mice following a reduction in TG6 protein levels.

## Discussion

Tranglutaminases (TGs) are a family of enzymes associated with the pathogenesis of neurodegenerative diseases, as first observed by Selkoe in the early 1980s, who noted their potential importance in protein cross-linking in Alzheimer’s disease (ad) [15]. Since then, TGs have been investigated in Huntington’s disease (HD) [16, 17], stroke [18], traumatic brain injury [19], and, more broadly, in processes leading to neuronal death [20]. Typically, their involvement has been associated with increased enzymatic activity. When intracellular calcium levels exceed 100 nM, TGs catalyze the transamidation of glutamine with amine donors. The amines may derive from lysines or from metabolites, such as polyamines and neurotransmitters, which have recently been shown to affect chromatin structure [21, 22]. Eventually, the amine in glutamine residues can be removed by deamination, and in rare cases, TGs can acylate lysines. Despite numerous studies published on TG biology, the mechanisms by which TGs contribute to neuronal damage remain incompletely understood. For example, we and others showed that TG2 modifies histone proteins when hyperactivated [17, 21], and increased TG1 activity has been associated with neuronal death in stroke and ad [18, 23]. Specific mutations of one isoform, TG6, have been implicated as causative in SCA35 [2]. However, the role of mutant TG6 in neurodegeneration, particularly in SCA35, remains unclear. At least two reports question a direct pathogenic role for TG6 in SCA35 [3, 4], while others provide supporting evidence [24, 25]. Finally, associations with Parkinson’s disease (PD) have also been proposed [26–28]. Nevertheless, all mutations considered pathogenic showed reduced enzymatic activity in in vitro models, suggesting that loss of TG6 function may underlie disease mechanisms.

Here, we show that Tgm6 < tm1a mice, which show significantly reduced *Tgm6* mRNA expression compared to WT mice, develop motor impairment beginning at 10 months of age. These findings provide strong evidence that TG6 loss-of-function contributes to motor deficits.

Importantly, motor alterations manifest only in male mice. The causes of this sex-bias are currently unknown and represent a limitation of our study. Our observation of *Tgm6* loss in female mice, coupled with a significant increase in *Tgm4,* suggests a sex-specific compensatory role. Of relevance, sex-specific prevalence is well-recognized in neurodegenerative conditions [29]. For instance, ad is common in females, whereas PD and Amyotrophic Lateral Sclerosis (ALS) are more prevalent in males, with reported male-to-female ratios of 2–4: 1.; however, clinical manifestations involving motor symptoms are more frequent in men [30, 31]. Our results, although preliminary, suggest that TG6 loss-of-function may represent a risk factor for motor diseases in males.

## Materials and methods

### Mice

C57BL/6 N-A^tm1Brd^ Tgm6^tm1a^(KOMP)^Wtsi/WtsiPh^ mice were purchased from The European Mouse Mutant Archive—EMMA. This mouse strain is characterized by a *Tgm6* gene knockout generated using the knockout-first allele strategy. Wild-type C57BL/6 mice were purchased from Charles River and used for mating with the transgenic animals. GLAST-Cre^ERT2^ transgenic mice that carry a tamoxifen-inducible form of Cre (Cre^ERT2^) expressed in the locus of the astrocyte-specific glutamate transporter (GLAST), were used for mating. Animal care and experimental procedures were performed in accordance with the Animal Welfare Body of the University of Trento and with the Italian Ministry of Health authorization (197/2019-PR). The mice were housed in groups of up to five per cage with free access to food and water. They were housed in IVC cages (Tecniplast GM500) with a relative humidity range of 20% to 70%, controlled temperature (20–22°C), and a 12-hour light–dark cycle. Body weight was measured weekly.

### Genotyping

Mouse ear tissues were punched out and used to genotype the animals. MyTaq™ Extract-PCR Kit was used according to the manufacturer’s instructions (Meridian Bioscience). PCR was performed with the following primer sets: Fwd *Tgm6* common 5′-CCCAGTGACAGCCACACAAG-3′; Rev *Tgm6* WT 5′-GCCTGAAAATTAGGG-CACTCTG-3′; Rev *Tgm6* transgene 5′-TCGTGGTATCGTTATGCGCC-3′.

### Behavioral tests

Every three months, mice were tested for the following behavioral tests: rotarod, ladder, and balance beam. Behavioral experiments were performed during the dark phase of the light–dark cycle in the presence of light.

#### Rotarod test

Mice were pre-trained the day before the test session; they were habituated to stay on the rotarod at a constant speed of 16 rpm. During the experiment, the animals run 3 consecutive trials at 16 rpm at a constant speed. The cut-off latency was 300 seconds, and 300 seconds of rest were provided between two trials. Latency to fall from the rotarod was measured in seconds, and we selected the best score across the three trials.

#### Ladder test

A 70 cm horizontal ladder was used to assess the motor coordination of the mice. The rungs, surrounded by two clear plexiglass side walls spaced 5 cm apart, were arranged in an irregular pattern that requires a skilled walk from the animals, which must navigate the ladder up to a black box. The same rung configuration was used to test all the animals at each time point. The apparatus was elevated horizontally 30 cm above the ground, and its end was placed on a black box with bedding. Animals were placed in a black box with their bedding for 5 minutes. After that, they were moved to their home cage and tested on the ladder one at a time. The black box was placed at the end of the ladder. The habituation consisted of walking over the ladder once. The test was performed immediately after. The performance was video recorded. All the video recordings were analyzed at a slower speed, and the total number of footfalls for all limbs was counted. An error was defined as a total miss of a step or a slipped contact.

#### Balance beam

The apparatus consists of a 70 cm beam with a flat surface measuring 8 mm in width. The beam was placed horizontally 30 cm above the ground. Animals were pre-trained on an apparatus with a 15 mm-wide beam. Immediately after, the mice were tested on an 8 mm beam. The time it took the mice to travel through the beam was measured in seconds. The maximum time assigned to each mouse when it fails the test is 60 seconds. The performance was video recorded from the side.

Animal performance during the behavioral tests (ladder and balance beam) was recorded using a Logitech C270 camera (721p/30fps) with OBS software and manually scored.

### RNA extraction

Total RNA was extracted using TRIzol (Life Technologies) according to the manufacturer’s instructions. RNA was resuspended in nuclease-free water, and its concentration was quantified using NanoDrop 2000 (ThermoFisher).

### Rt-PCR

A total of 1 μg of RNA was retrotranscribed using LunaScript® RT SuperMix Kit (E3010, New England Biolabs) according to the manufacturer’s instructions. Quantitative PCR (qPCR) was performed using ExcelTaq™ 2X Fast Q-PCR Master Mix (TQ1200, SMOBIO). Each sample was analyzed in triplicate for each gene. The ΔCt analysis was applied to the raw data, with normalization performed on *Actb* as the housekeeping gene. The results were then relativized to the levels of WT mice. qPCR was performed with the following primer sets: Fwd *Tgm1* 5′-TGTGGAGATCCTGCTCAGCTACCTA-3′; Rev *Tgm1* 5′-TGTCTGTGTCGTGTGCAGAGTTGA-3′; Fwd *Tgm2* 5′-TTCCGGCTGACTCTGTACTTCGAG-3′; Rev *Tgm2* 5′-ACATTGTCCTGTTGG-TCCAGCACT-3′; Fwd *Tgm3 5′-*GTGGTTGCAAGCGGATGATGTCTT-3′; Rev *Tgm3* 5′-AGTTCCAGCCAACCATGCCAATTC-3′; Fwd *Tgm4* 5′-TCTCCAACCTCAGGATGAGCTGAA-3′; Rev *Tgm4* 5′-TGTCACTGGATCAA-GCTCCACCAT-3′; Fwd *Tgm5* 5′-ATCAGCACCAAGAGCATCCAGAGT-3′; Rev *Tgm5* 5′-TGCAGAGCCTTTAGGAACACCTCT-3′; Fwd *Tgm6* 5′-TGTCACCAGGATCATCAGTGCCAT-3′; Rev *Tgm6* 5′-TGGTACCACCTCCA-TATTTGCCCT-3′; Fwd *F13A* 5′-TGTATGTTGCAGTCTGGACTCCCT-3′; Rev F13A 5′-TACACAGCGTCCTCTTCACACCAA-3′; Fwd *Actb* 5′-GACAGGAT-GCAGAAGGAGATTACTG-3′; Rev *Actb* 5′-CTCAGGAGGAGCAATGATCTT-GAT-3′.

### Immunofluorescence

Animals were anesthetized and perfused with 0.1 M phosphate-buffered saline (PBS, pH 7.4) followed by 4% paraformaldehyde (PFA) in PBS. Brains were collected and post-fixed in 4% PFA overnight, followed by dehydration in 70% ethanol and paraffin-embedding. Sagittal sections were obtained using a standard rotary microtome (Leica HistoCore Biocut Microtome) and deparaffinized in xylene. Heat-induced antigen unmasking using tris-EDTA (pH 8) was performed before blocking for 1 hour in 5% Fetal Bovine Serum (FBS). Finally, brain slices were incubated overnight with primary antibodies, followed by a 1-hour incubation with secondary antibodies the next day. Images were acquired using a Leica TCS SP8 laser-scanning confocal microscope equipped with an HC PL APO 20x/0.75 Air CS2 objective. TG6 Mean Intensity was measured with Fiji (ImageJ 1.53 t), using a custom-written macro that quantifies the signal in the area in the SUM projection of the acquired z-stack of the Calbindin^+^ neurons.

Primary antibodies include anti-Calbindin (1:500, Sigma Aldrich, C9848), and TG6 (1:100, Zedira, A017). Primary antibodies were detected using donkey anti-rabbit IgG, Alexa Fluor 488 (1:500, ThermoFisher Scientific, A21206) and goat anti-mouse IgG, Alexa Fluor 647 (1:500, ThermoFisher Scientific, A21235). Nuclei were stained with Hoechst (2ug/ml, Sigma Aldrich, B2261).

## Data analysis

All the data were collected and reorganized in Excel files, then exported to GraphPad Prism (v 11.0.0). Statistics were performed using the unpaired two-tailed Student’s *t-*test, Welch’s *t-*test, or Kruskal-Wallis test with Dunn’s post-hoc multiple comparisons. For body weight, mixed-effect analysis with Tukey’s multiple comparisons test was performed. Error bars in all graphs are presented as the standard error of the mean (SEM).

## Supplementary Material

Supplemental_Data_(web_posting_only)_ddag037

## Data Availability

Upon request to manuela.basso@unitn.it.
