# Supplementary material for: Partial TG6 loss of function causes motor deficits in male mice
Source: Hum Mol Genet. 2026 Jun 4;35(10):ddag037. doi: 10.1093/hmg/ddag037 (PMC13235716; doi:10.1093/hmg/ddag037)
Supplement: Supplemental_Data_(web_posting_only)_ddag037 [file supplemental_data_(web_posting_only)_ddag037.pdf]

Supplemental Data

Partial TG6 loss of function causes motor deficits in male mice

Luisa Donini[1], Linda Sartori[1], Anna Barbieri[1], Alice Migazzi[1], Sergio Robbiati[1,2], Maria Pennuto[3,4], Manuela Basso[1*]

[1] Department of Cellular, Computational and Integrative Biology (CIBIO), University of Trento, Trento, Italy.
[2] Model Organism Core Facility (MOF), Department CIBIO, University of Trento, Trento, Italy.
[4] Department of Biomedical Sciences (DBS), University of Padova, 35131 Padova, Italy
[5] Veneto Institute of Molecular Medicine (VIMM), 35129 Padova, Italy

*For correspondence: manuela.basso@unitn.it

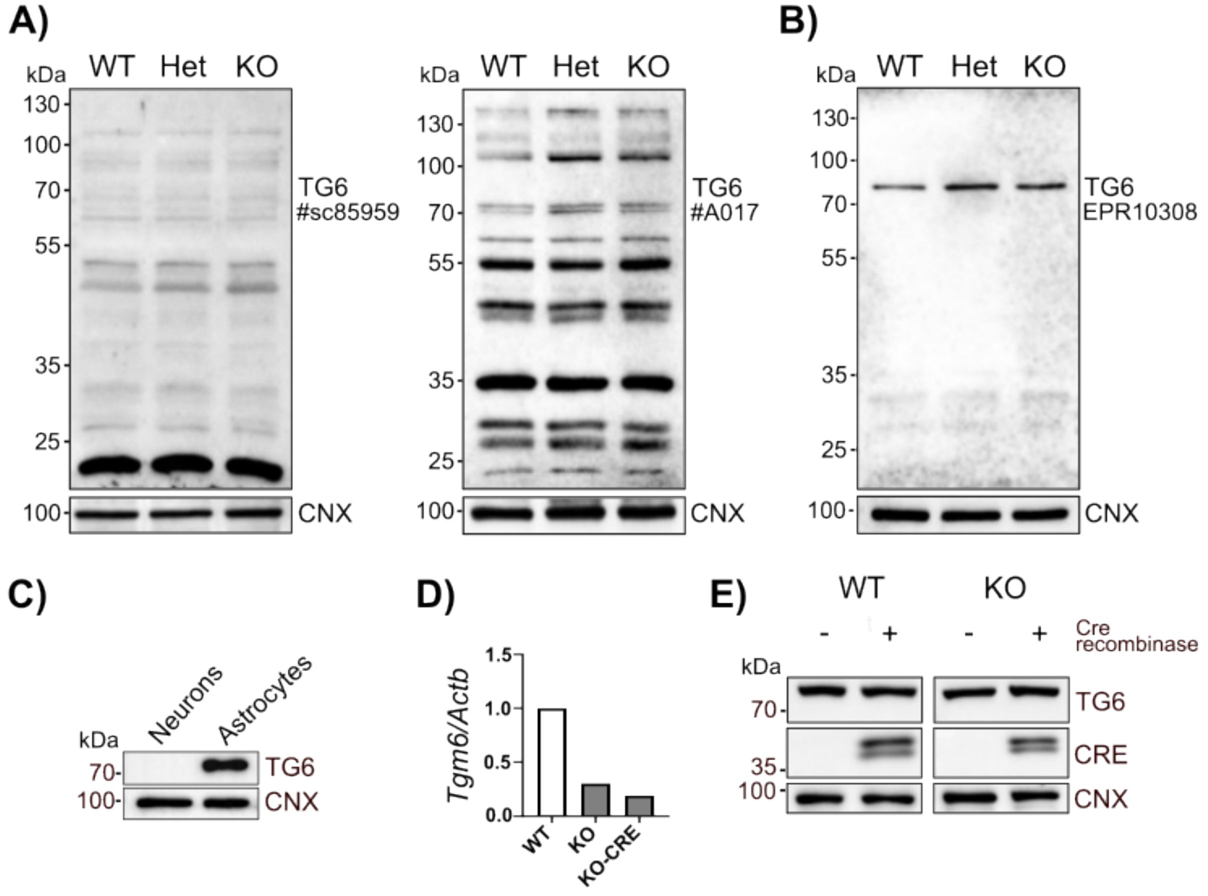

Supplementary Figure 1. Detection of TG6 in immunoblotting.
(**A**) Representative immunoblotting of TG6, performed with antibodies #sc-85959 and #A017, on cerebellum lysates from TG6 WT/-Het/-KO animals. Calnexin is reported to confirm equal loading. (**B**) Representative immunoblotting of TG6 (antibody clone EPR10308) on cerebellum lysates from Tgm6-WT/-Het/-KO animals. (**C**) Representative immunoblotting of TG6 (antibody clone EPR10308) in WT primary neurons and astrocytes. (**D**) RT-PCR of the *Tgm6* transcript normalized on *Actb* in WT and TG6 KO primary cortical astrocytes expressing the Cre recombinase (n=1). (**E**) Representative immunoblotting of TG6 protein (antibody clone EPR10308) in TG6-WT and TG6 KO primary cortical astrocytes expressing the Cre recombinase (n=1).

Supplementary Materials and Methods

Protein extraction

Tissue powder was lysed by homogenizing in RIPA solution (Tris-HCl, pH 8.0, 50 mM NaCl, 150 mM NaCl, 1% NP-40, 0.5% sodium deoxycholate, and 2% SDS) and then supplemented with a protease inhibitor cocktail (P8340, Sigma-Aldrich). After nuclease treatment, the samples were centrifuged at 4°C for 15 minutes at 21.000 g. Finally, the supernatant of each sample was collected and preserved at -80°C.

Total protein concentration was determined using the DC Protein Assay, according to the manufacturer's instructions (Bio-Rad). Absorbance was read at 750nm by Varioskan™ LUX Multimode Microplate Reader (Thermo Scientific).

Protein immunoblotting

Lysates were run in SDS-PAGE under reducing conditions after being denatured at 95°C for 5 minutes. Gels were wet transferred using 20% methanol onto nitrocellulose membrane at 4°C and subsequently blocked in blocking buffer (5% non-fat milk, 0,1% Tween-20 in TBS buffer). Primary antibodies were diluted in blocking buffer and incubated overnight at 4°C. Three washes were performed in 0,1% Tween-20 in TBS buffer (TBST) before the addition of HRP-conjugated secondary antibodies diluted 1:10.000 in blocking buffer, for 1 hour at room temperature. Three washes were performed in TBST before image acquisition after incubation with ECL™ Select Western Blotting Detection Reagents (Cytiva Amersham) on the Bio-Rad ChemiDoc imaging system. The primary antibodies used are anti-TG6 (1:1000, Abcam ab180959, EPR10308), anti-TG6 (1:1000, Zedira, A017), anti-TG6 (1:1000, Santa Cruz Biotechnology, sc-85959), anti-Calnexin (1:1000, Enzo, ADI-SPA-860-F), and anti-Cre (1:1000, Biolegend, 908001).

Primary cortical neurons and astrocytes

Primary cortical neurons and astrocytes were obtained from mice at embryonic stage E15.5 or pups at P0-2, respectively. Cortices were digested in papain solution (20U papain, 500 µM EDTA, 100 µM Cystine, 26 mM Sodium Bicarbonate in 1X Earle's Balanced Salt Solution (EBSS, Gibco)) at 37°C for 20 minutes. After DNase I treatment at 37°C for 3 minutes, they were centrifuged at 1500 x g for 5 minutes and resuspended in EBSS supplemented with Bovine Serum Albumin and Trypsin Inhibitor. Cells were centrifuged at 1500 x g for 10 minutes and resuspended in the plating medium (for neurons: 10% FBS, 1% Pen Strep in MEM with L-Glutamine; for astrocytes: 10% FBS, 1% L-Glutamine, 1% Pen Strep in DMEM). Cortical neurons were seeded on pre-coated wells with poly-D-lysine (Sigma). The day after, and at 7 days in vitro (DIV), the whole medium was replaced with Neurobasal medium supplemented with 1% B27, 1 mM Sodium Pyruvate, 1% Penicillin-Streptomycin, 2 mM L-glutamine, and 8 µM AraC (Sigma) for neurons, and with plating medium for astrocytes. Cells were cultured at 37°C with 5% CO_2.

Astrocyte transduction

For lentiviral vector production, the plasmids for Cre recombinase (Addgene #12238**),** psPAX2 plasmid (Addgene #12260) containing *gag*, *pol,* and *rev* genes, and VSV-G envelope plasmid

(pMD2.G plasmid, Addgene #12259) were transfected into HEK293T cells by calcium phosphate. The whole medium was changed 16 hours after transfection, and 24 hours later, the medium was collected, centrifuged at 1200 x g for 10 minutes, 0.45 μm filtered, and stored at -80 °C. Before transduction, the viruses were quantified using the SG-PERT reverse assay as previously described (1). Mouse primary astrocytes were transduced at DIV5 with a multiplicity of infection (MOI) of 10. The day after transduction, the whole medium was replaced with fresh medium. Cells were collected at DIV15.

References

1. Tripathy, D., Vignoli, B., Ramesh, N., Polanco, M.J., Coutelier, M., Stephen, C.D., Canossa, M., Monin, M.-L., Aeschlimann, P., Turberville, S., *et al.* (2017) Mutations in TGM6 induce the unfolded protein response in SCA35. *Human Molecular Genetics*, **26**, 3749–3762.
